# Probability Representation of Quantum States

**DOI:** 10.3390/e23050549

**Published:** 2021-04-29

**Authors:** Olga V. Man’ko, Vladimir I. Man’ko

**Affiliations:** 1 Lebedev Physical Institute, Russian Academy of Sciences, Leninskii Prospect 53, 119991 Moscow, Russia; mankoov@lebedev.ru; 2 Moscow Institute of Physics and Technology, State University, Institutskii per. 9, Dolgoprudnyi, 141700 Moscow, Russia; 3 Russian Quantum Center, Skolkovo, 143025 Moscow, Russia; 4 Department of Physics, Tomsk State University, Lenin Avenue 36, 634050 Tomsk, Russia

**Keywords:** probability distribution, tomography, quantizer, dequantizer, star–product, qubit

## Abstract

The review of new formulation of conventional quantum mechanics where the quantum states are identified with probability distributions is presented. The invertible map of density operators and wave functions onto the probability distributions describing the quantum states in quantum mechanics is constructed both for systems with continuous variables and systems with discrete variables by using the Born’s rule and recently suggested method of dequantizer–quantizer operators. Examples of discussed probability representations of qubits (spin-1/2, two-level atoms), harmonic oscillator and free particle are studied in detail. Schrödinger and von Neumann equations, as well as equations for the evolution of open systems, are written in the form of linear classical–like equations for the probability distributions determining the quantum system states. Relations to phase–space representation of quantum states (Wigner functions) with quantum tomography and classical mechanics are elucidated.

## 1. Introduction

The main goal of the paper is to give a review of conventional quantum mechanics in which the states of quantum systems are identified with probability distributions both for physical systems with continuous variables, like free particles and oscillators, as well as for systems with discrete variables, like spins. In quantum mechanics, the system states (e.g., oscillator, spin-1/2 particle, hydrogen atom, photon states) are described by wave functions [1], density matrices, and density operators [2,3] acting in the Hilbert spaces of the state vectors [4]. The quantum observables are associated with Hermitian operators acting in these spaces. The notion of quantum states associated with Hilbert space vectors and density operators is very different from the notion of states in classical mechanics and classical statistical mechanics.

The discussion of the attempts to give the probability formulation of quantum states has long history. We mention the work of Wooters [5] where the attempt to find the formulation of quantum state where instead of probability amplitude (wave function) “the probability table” was discussed for spin-1/2 system.

Attempts to find the state representation for systems with continuous variables like position more similar to the system state notions used in classical mechanics and in classical statistical mechanics were suggested in References [6,7,8,9]. For discrete spin variables, there were also suggestions by Stratonovich to introduce analogous representations [10] using method of quantizer operators. In connection with experimental results, to use the quantum optical tomography to measure the Wigner functions of photon states [11], the introducing of the notion of symplectic tomographic probability distribution was applied to associate the quantum states with this probability distributions [12]. For discrete variables of spin-1/2 systems, the probability description of system states was suggested in References [13,14]. We consider the general construction [12,15] for arbitrary quantum systems, where the density operators are invertibly mapped onto probability distributions including the equations for energy levels and evolution equations for quantum states. We present the construction for three paradigmatic examples—harmonic oscillator, free particle, and spin-1/2 (qubit system). We will show that free particle states and their evolutions can be presented in the form of tomographic probability distributions of coherent states introduced for oscillator in References [8,9,16].

The probability distributions are standard normal distributions invertibly mapped onto density matrices of the free particle states. The normalized Fock state tomograms of free particle are also explicitly constructed using method of time dependent integrals of motion [17]. The symplectic tomographic probability distribution was introduces for description of quantum states since its partial case (optical tomography technique [18,19]) was used to obtain the photon quantum states identified with the Wigner function [6] in quantum optics experiments [11]. For spin system with discrete variables, the Wigner function introduced in Reference [10] and the possibility to map this Wigner function onto the spin probability distribution was suggested in References [14,20]. The nonredundant tomographic description of spin-1/2 states corresponding to “probability table” [5] was presented in References [15,21].

The main aim of the paper is to review the methods of obtaining the probability representation of quantum states and to formulate the rules of constructing the probability distribution representations of quantum system states on examples of the quantum oscillator, free particle and qubit. The idea of this construction is to find the invertible map of density operators onto probability distributions applying the Born’s rule [22,23] and the method of quantizer–dequantizer operators [24,25,26] (also see References [27,28]). The development and review of this method of quantizer–dequantizer, which is important for studying the systems with continuous variables is presented in Reference [29]. The problem of the probabilities used in quantum mechanics was discussed, for example, in Reference [30]. The consideration of nonnegative probabilities in quantum mechanics was discussed in References [31,32]. In Reference [32], it was shown that key concepts of classical mechanics can be reformulated in the language of Hermitian operators using the inverse Wigner-Weyl transform of the classical probability distributions. In Reference [33], the attempts to study the problems of complex biosystems composed of many subsystems using the language of quantum mechanics and thermodynamics were done. In Reference [34], a quantum mechanical analysis of Bell’s approach to quantum foundations based on his hidden-variable model was presented. In Reference [35], a review of classical probability representations of quantum states and observables is done. In Reference [36], the application of quantum information and field theories to modeling of social process is done. In Reference [37], the probability domain was extended to the complex space, and the relation of the complex probabilities to the quantum probabilities was obtained. The relation between the quantum state description and the classical state description is elucidated in References [26,38,39,40]. In Reference [41], a tomographic representation of quantum cosmology was considered. The extension of the tomographic maps to the quantum case and a Weyl-Wigner quantization in the classical case were considered in Reference [42]. The methods of star-product, tomography, and probability representation of quantum mechanics were applied to different problems of quantum phenomena in References [43,44,45,46,47,48]

In this paper, we use only conventional nonnegative probabilities. We will use such probabilities to describe evolution equations of quantum mechanics—Schrodinger equation [1], von Neumann equation [3], the Gorini–Kossakowski–Sudarshan–Lindblad (GKSL) equation [49,50,51]. Quantum evolution equation for open system (GKSL) was considered in References [52,53]. Different kinds of equations including the quantum evolution equation were considered in References [54,55]. Applications of tomographic probability representation in quantum information processing were discussed in Reference [56]. Applications of quantum tomographic approach to different kinds of experiments, as well as theoretical research studies, were discussed in References [57,58,59,60,61]. The tomograms and eigenvalues of energy computed in terms of tomographic symbols are shown in Reference [62]. The dynamics of quantum particles was described by Kolmogorov equations for nonnegative propagators in tomography representation in Reference [63].

The paper is organized as follows. In Section 2, the symplectic tomographic probability representation for the quantum and classical oscillator is discussed. In Section 3, the general scheme of constructing the probability representation of quantum states by means of quantizer–dequantizer is presented. In Section 4, an associative product of the operator symbols is considered. In Section 5, the free particle tomography of the coherent and the Fock states are discussed. The equation of quantum mechanics is given in Section 6. In Section 7, qubit states are considered in probability representation of quantum mechanics. In Section 8, the probability representation of qudit state is introduced using quantizer–dequantizer method. It is necessary to add that there may exist other easier methods to achieve analogous result. In Section 9, quantum evolution of open system in probability representation of density operator is discussed. The conclusions are presented in Section 10.

## 2. Probability Representation of Quantum Oscillator States

The conventional formulation of harmonic oscillator state theory is based on the notion of wave function ψ(x,t) of the oscillator which obeys the quantum evolution Schrödinger equation (Planck constant and oscillator mass we take equal to unity):

(1) $$i\frac{\partial \psi (x,t)}{\partial t}=-\frac{1}{2}\frac{\partial^{2}\psi \left(x,t\right)}{\partial x^{2}}+\frac{\omega^{2}x^{2}}{2}\psi \left(x,t\right).$$

For frequency of the oscillator ω=0, we have the Schrödinger equation for the quantum free particle. The complex wave function ψ(x,t)=|ψ(x,t)|exp(iϕ(x,t)) for stationary states of the harmonic oscillator satisfies the eigenvalue equation for the Hamiltonian $\hat{H}=\frac{{\hat{p}}^{2}}{2}+\frac{\omega^{2}{\hat{q}}^{2}}{2}$. The state vectors $|\psi_{n}\rangle ,\;n=0,1,2,\ldots$, of the stationary states of the harmonic oscillator obey to the Schrödinger equation:

(2) $$\hat{H}|\psi_{n}\rangle =E_{n}|\psi_{n}\rangle ,\;n=0,1,2,\ldots .,$$

which has the solutions for the Fock state wave functions $\psi_{n}\left(x\right)=\langle x|\psi_{n}\rangle$ of the form

(3) $$\psi_{n}\left(x\right)=\frac{e^{-\frac{x^{2}}{2}}}{\pi^{1/4}}\frac{1}{\sqrt{2^{n}n!}}H_{n}\left(x\right),\;E_{n}=\left(n+1/2\right),$$

where $H_{n}\left(x\right)$ is Hermite polynomial (see books on quantum mechanics [64,65]). The density operator ${\hat{\rho }}_{n}$ of the Fock state of the harmonic oscillator satisfies the equation

(4) $$\hat{H}{\hat{\rho }}_{n}=E_{n}{\hat{\rho }}_{n}.$$

We address the following problem. Is it possible to find an invertible map of the density operators of the Fock states ${\hat{\rho }}_{n}$ and density operators $\hat{\rho }$ of any other states of the harmonic oscillator onto probability distributions? Such maps are described by the existing pairs of quantizer–dequantizer operators denoted for dequantizer as $\hat{U}\left(\vec{x}\right)$ and for quantizer as $\hat{D}\left(\vec{x}\right)$, $\left(\vec{x}=X,\mu ,\nu \right)$ [24]. Here, we introduce three real continuous parameters −∞<X,μ,ν<∞. If we take

(5) $$\hat{U}\left(X,\mu ,\nu \right)=\delta \left(X\hat{1}-\mu \hat{q}-\nu \hat{p}\right),\;\hat{D}\left(X,\mu ,\nu \right)=\frac{1}{2\pi }\exp\left[i\left(X\hat{1}-\mu \hat{q}-\nu \hat{p}\right)\right],$$

the invertible map of the density operator $\hat{\rho }$ onto the function ω(X|μ,ν) reads [12]

(6) $$\begin{array}{c} \omega \left(X|\mu ,\nu \right)=Tr\left[\hat{\rho }\delta \left(X\hat{1}-\mu \hat{q}-\nu \hat{p}\right)\right],\\ \hat{\rho }=\frac{1}{2\pi }\int \omega \left(X|\mu ,\nu \right)\exp\left[i\left(X\hat{1}-\mu \hat{q}-\nu \hat{p}\right)\right]dXd\mu d\nu . \end{array}$$

The function ω(X|μ,ν) called symplectic tomogram is nonnegative probability distribution function of the random position *X* satisfying the normalization condition

(7) ∫ω(X|μ,ν)dX=1.

The tomogram of Fock states (ω=1) can be found, and it has the form

(8) $$\omega_{n}\left(X|\mu ,\nu \right)=\frac{e^{-\frac{X^{2}}{\left(\mu^{2}+\nu^{2}\right)}}}{\sqrt{\pi (\mu^{2}+\nu^{2})}}\frac{1}{2^{n}n!}H_{n}^{2}\left(\frac{X}{\sqrt{\mu^{2}+\nu^{2}}}\right).$$

Using (6) for pure state $\hat{\rho }=\left|\psi \rangle \langle \psi \right|$, one can express the tomographic probability distribution w(X|μ,ν) in terms of wave function ψ(y) of system [66]

(9) $$w\left(X|\mu ,\nu \right)=\frac{1}{2\pi |\nu |}{\left|\int \psi \left(y\right)e^{\frac{i\mu }{2\nu }y^{2}-\frac{ixy}{\nu }}dy\right|}^{2}.$$

Thus, we map the wave function ψ(y) onto probability distribution w(X|μ,ν) of random position *X*. The physical meaning of the parameters μ and ν can be clarified if we consider symplectic tomogram for the classical oscillator state with the probability density f(q,p) of the state of the system. Namely, the position of the oscillator can be measured in different reference frames of classical particle in the phase space. The position *X* is the position in transformed reference frame. If *q* and *p* are position and momentum in initial reference frame, and we introduce the variables $q^{\prime \prime }=sq$, $p^{\prime \prime }=s^{-1}p$ and $q^{\prime }=cos\theta q^{\prime \prime }+\sin\theta p^{\prime \prime }$, $p^{\prime }=cos\theta p^{\prime \prime }-\sin\theta q^{\prime \prime }$, where *s* is scaling parameter, then *X* is position measured in the new reference frame with parameters $q^{\prime }$ and $p^{\prime }$. Thus, the position X=μq+νp is the position measured in scaled (parameter *s*) and rotated (parameter θ) reference frame. Obviously, one has the marginal probability distribution

(10) w(X|μ,ν)=∫f(q,p)δ(X−μq−νp)dqdp,

given by Radon transform [67] and the inverse transform reads

(11) $$f\left(q,p\right)=\frac{1}{4\pi^{2}}\int w\left(X|\mu ,\nu \right)e^{i(X-\mu q-\nu p)}dX\;d\mu \;d\nu .$$

In classical mechanics, the probability density $f\left(q,p\right)=\delta \left(q-q_{0}\right)\delta \left(p-p_{0}\right)$ of the state without fluctuations of the position and momentum describes the classical particle state with given position $q_{0}$ and momentum $p_{0}$. The tomogram of such state $w_{0}\left(X|\mu ,\nu \right)=\delta \left(X-\mu q_{0}-\nu p_{0}\right)$ describes such classical particle state. It does not exist as tomogram of a real quantum particle in view of uncertainty relation [68]. For classical pendulum (harmonic oscillator with m=ω=1), at the temperature *T*, the probability density $f\left(q,p\right)=\frac{1}{2\pi T}\exp\left[-(q^{2}+p^{2})/2T\right]$. For temperature T→0, the $f_{T}\left(q,p\right)\to \delta \left(q\right)\delta \left(p\right)$. It means that the tomogram, which is Gaussian probability distribution for very small temperature, violates the Heisenberg uncertainty relation for position and momentum.

## 3. General Scheme of Using Quantizer–Dequantizer Formalism

Let us discuss the problem of constructing invertible maps of different kinds of any operator $\hat{A}$ acting in a Hilbert space H of Dirac vectors |ψ〉 onto function $f_{A}\left(\vec{x}\right)$ called symbol of operator $\hat{A}$, where $\left(\vec{x}\right)=\left(x_{1},x_{2},\ldots ,x_{N}\right)$ have components either discrete or continuous $x_{j}$, j=1,2,…,N. It means that we can find the pairs of operators $U(\vec{x})$, $\hat{D}\left(\vec{x}\right)$ such that

(12) $$f_{\hat{A}}\left(\vec{x}\right)=\mathrm{Tr}\hat{A}\hat{U}\left(\vec{x}\right),\;\hat{A}=\int f_{\hat{A}}\left(\vec{x}\right)\hat{D}\left(\vec{x}\right)d\vec{x},$$

in the case of continuous parameters $x_{j}$. The integral is replaced by corresponding sum in case of discrete parameters $x_{j}$. Equation (12) is a generalization of concrete formulas (5), where the concrete case of symplectic tomographic probability representation of the oscillator states was discussed. The idea of using the map $\hat{A}↔f_{\hat{A}}\left(\vec{x}\right)$ is related to possibility to replace the formalism of using operators by the formalism of using the functions. In quantum mechanics, it is realized by phase–space representation of quantum states [69], e.g., by description of the quantum oscillator state by the Wigner function W(q,p) [6]. In the case of the map of the oscillator state density operator $\hat{\rho }$ onto the Wigner function, the dequantizer $\hat{U}\left(\vec{x}\right)$, where $\vec{x}=\left(x_{1},x_{2}\right)=\left(q,p\right)$, has the form

(13) $$\hat{U}\left(q,p\right)=\int |q-u/2\rangle \langle q+u/2|e^{-ipu}du,\;\hbar =m=\omega =1$$

and quantizer operator $\hat{D}\left(q,p\right)={\left(2\pi \right)}^{-1}\hat{U}\left(q,p\right)$. Here, the vector |q+u/2〉 is eigenvector of the position operator $\hat{q}$, i.e., $\hat{q}|q+u/2\rangle =\left(q+u/2\right)|q+u/2\rangle$. For density operator of pure state ${\hat{\rho }}_{\psi }=\left|\psi \rangle \langle \psi \right|$, the Wigner function of the oscillator is expressed in terms of the wave function as follows:

(14) $$W\left(q,p\right)=\int \psi \left(q+u/2\right)\psi^{*}\left(q-u/2\right)e^{-ipu}du,$$

and

(15) $$\psi \left(x\right)\psi^{*}\left(x^{\prime }\right)=\frac{1}{2\pi }\int W\left(\frac{x+x^{\prime }}{2},p\right)e^{ip(x-x^{\prime })}dp.$$

The Wigner function is expressed in terms of dequantizer operator (13) and density operator $\hat{\rho }$, and it is the Wigner–Weyl symbol of the state density operator. Thus, we have the possibility to introduce the description of the oscillator state with density operator $\hat{\rho }$ either by symplectic tomogram w(X|μ,ν) or by the Wigner function W(q,p). The expression of the tomogram in terms of the Wigner function reads

(16) $$w\left(X|\mu ,\nu \right)=\int W\left(q,p\right)\delta \left(X-\mu q-\nu p\right)\frac{dq\;dp}{2\pi }.$$

The Wigner function is expressed in terms of the tomogram as

(17) $$W\left(q,p\right)=\frac{1}{2\pi }\int w\left(X|\mu ,\nu \right)e^{i(X-\mu q-\nu p)}dX\;d\mu \;d\nu .$$

For some states, the real Wigner function takes negative values. For example, odd first excited states $|1\rangle ={\hat{a}}^{†}|0\rangle$ of the oscillator and higher excited odd states $|2k+1\rangle ={\left(\left(2k+1\right)!\right)}^{-1/2}{\left({\hat{a}}^{†}\right)}^{2k+1}|0\rangle$, k=0,1,2,…,∞ are odd continuous wave functions. The Wigner functions of these states satisfy the condition

(18) $$W_{2k+1}\left(0,0\right)=\int \psi_{2k+1}\left(u/2\right)\psi_{2k+1}^{*}\left(-u/2\right)\delta \left(u\right)\;du=0,$$

since these states have the property $\psi_{\left(2k+1\right)}\left(0\right)=0.$ The symplectic tomogram w(X|μ,ν)≥0. This tomogram for μ=cosθ, ν=sinθ equals to optical tomogram [18,19], which determines the Wigner function

(19) $$w\left(X|\cos\theta ,\sin\theta \right)=w_{opt}\left(X|\theta \right)=\int W\left(q,p\right)\delta \left(X-q\cos\theta -p\sin\theta \right)\frac{dq\;dp}{2\pi }.$$

In view of the property $\delta \left(\lambda x\right)=\frac{1}{\left|\lambda \right|}\delta \left(x\right)$, one has the relation

(20) $$w\left(X|\mu ,\nu \right)=\frac{1}{\sqrt{\mu^{2}+\nu^{2}}}w_{opt}\left(X|\theta =\arctan\frac{\nu }{\mu }\right).$$

The optical tomogram of photon state is measured in experiments with homodyne detector [11]. It means that the Wigner function can be reconstructed using Equations (17) and (20). Since the tomogram which is experimentally measured contained complete information on the photon quantum states it was suggested [12] to identify the state with tomographic probability distribution function interpreting it as primary notion of quantum state and calling this method as probability representation of quantum states.

## 4. Associative Product of the Operator Symbols

The operators $\hat{A}$, $\hat{B}$ describing the quantum observables can be multiplied. This multiplication has the property of associativity, i.e., $\left(\hat{A}\hat{B}\right)\hat{C}=\hat{A}\left(\hat{B}\hat{C}\right)$. Symbols of the operators, i.e., the functions $f_{\hat{A}}\left(\vec{x}\right)$, $f_{\hat{B}}\left(\vec{x}\right)$, $f_{\hat{C}}\left(\vec{x}\right)$ have to satisfy the property of the associativity. It means that the associative product of the functions called star–product $\left(f_{\hat{A}}★f_{\hat{B}}\right)\left(\vec{x}\right)=f_{\hat{A}\hat{B}}\left(\vec{x}\right)$ is nonlocal product, and it must satisfy the condition

(21) $$\left(\left(f_{\hat{A}}★f_{\hat{B}}\right)★f_{\hat{C}}\right)\left(\vec{x}\right)=\left(f_{\hat{A}}★\left(f_{\hat{B}}★f_{\hat{C}}\right)\right)\left(\vec{x}\right).$$

The nonlocal star–product is determined by the integral kernel $K({\vec{x}}_{1},{\vec{x}}_{2},{\vec{x}}_{3})$, i.e.,

(22) $$\left(f_{\hat{A}}★f_{\hat{B}}\right)\left({\vec{x}}_{3}\right)=\int K\left({\vec{x}}_{1},{\vec{x}}_{2},{\vec{x}}_{3}\right)f_{\hat{A}}\left({\vec{x}}_{1}\right)f_{\hat{B}}\left({\vec{x}}_{2}\right)d{\vec{x}}_{1}\;d{\vec{x}}_{2}.$$

One can see that this kernel is determined by dequantizer $\hat{U}\left(\vec{x}\right)$ and quantizer $\hat{D}\left(\vec{x}\right)$ operators

(23) $$K\left({\vec{x}}_{1},{\vec{x}}_{2},{\vec{x}}_{3}\right)=\mathrm{Tr}\left(\hat{D}\left({\vec{x}}_{1}\right)\hat{D}\left({\vec{x}}_{2}\right)\hat{U}\left({\vec{x}}_{3}\right)\right).$$

For a system with continuous variables, like harmonic oscillator (m=ω=ℏ=1), the kernel is equal to the function

(24) $$\begin{array}{ccc} & & K\left(X_{1},X_{2},X_{3},\mu_{1},\mu_{2},\mu_{3},\nu_{1},\nu_{2},\nu_{3}\right)=\frac{1}{4\pi }\delta \left[\mu_{3}\left(\nu_{1}+\nu_{2}\right)-\nu_{3}\left(\mu_{1}+\mu_{2}\right)\right]\\ & & \times \exp\left[\frac{i}{2}\left(\nu_{1}\mu_{2}-\nu_{2}\mu_{1}+2X_{1}+2X_{2}-2\frac{\nu_{1}+\nu_{2}}{\nu_{3}}X_{3}\right)\right]. \end{array}$$

For the oscillator, the associative product of Wigner–Weyl symbols, e.g., star–product of Wigner functions, is determined by the Grönewold kernel [70]

(25) $$K\left(q_{1},p_{1},q_{2},p_{2},q_{3},p_{3}\right)=\frac{1}{4\pi^{2}}\exp\left[2i\left(q_{1}p_{2}-q_{2}p_{1}\right)+\left(q_{2}p_{3}-q_{3}p_{2}\right)+\left(q_{3}p_{1}-q_{1}p_{3}\right)\right],$$

which is given by (23), where dequantizer operators $\hat{U}\left(q,p\right)$ is given by (13) and quantizer $\hat{D}\left(q,p\right)=\frac{1}{2\pi }\hat{U}\left(q,p\right)$. From the relation $\hat{A}=\int f_{\hat{A}}\left(\vec{x}\right)\hat{D}\left(\vec{x}\right)d\vec{x}=\int f_{\hat{A}}\left(\vec{y}\right){\hat{D}}^{\prime }\left(\vec{y}\right)d\vec{y}$, where $\hat{D}\left(\vec{x}\right)$, $\hat{U}\left(\vec{x}\right)$ and ${\hat{D}}^{\prime }\left(\vec{y}\right)$, ${\hat{U}}^{\prime }\left(\vec{y}\right)$ are two different pairs of quantizer–dequantizer operators, we obtain the relation which connects two different symbols of the same operator $\hat{A}$

(26) $$f_{\hat{A}}\left(\vec{y}\right)=\int f_{\hat{A}}\left(\vec{x}\right)\mathrm{Tr}\left({\hat{U}}^{\prime }\left(\vec{y}\right)\hat{D}\left(\vec{x}\right)\right)d\vec{x}.$$

In the case of relation of the tomographic quantizer–dequantizer operators with the Wigner–Weyl symbols, the Formula (26) yields the connection of the state Wigner function W(q,p) with the symplectic tomogram (16), (17). For the Wigner function and the symplectic tomogram, the associative product was studied in Reference [71]. The review of the associative product is given in Reference [72]. These relations are the Radon transform relations analogous to (10), (11) used to connect the tomogram of classical oscillator states associated with probability density f(q,p) with classical symplectic tomography. The difference of classical and quantum symplectic tomograms is connected with the property of the probability distribution f(q,p) of the classical oscillator which is permitted by the Heisenberg uncertainty inequality. The Wigner function W(q,p) can take negative values, but the probability density f(q,p) must be nonnegative. But nonnegative densities f(q,p) with very small both dispersions of position and momentum after Radon transform provide the tomograms corresponding to related density operators $\hat{\rho }$ with negative eigenvalues.

## 5. Free Particle Coherent States in the Probability Representation of Quantum Mechanics

In this section, we consider the motion of free particle with the Hamiltonian $\hat{H}={\hat{p}}^{2}/2$ as the motion of the harmonic oscillator in the limit of zero frequency ω=0. One can check that the quantum free particle has two integrals of motion

(27) $${\hat{q}}_{0}\left(t\right)=\hat{q}-\hat{p}t,\;{\hat{p}}_{0}\left(t\right)=\hat{p}.$$

The integrals of motion of the free particle satisfy the equation

(28) $$\begin{array}{ccc} & & \frac{d{\hat{q}}_{0}\left(t\right)}{dt}=\frac{\partial {\hat{q}}_{0}\left(t\right)}{\partial t}+i\left[\hat{H},{\hat{q}}_{0}\left(t\right)\right]=0,\\ & & \frac{d{\hat{p}}_{0}\left(t\right)}{dt}=\frac{\partial {\hat{p}}_{0}\left(t\right)}{\partial t}+i\left[\hat{H},{\hat{p}}_{0}\left(t\right)\right]=0, \end{array}$$

and ${\hat{q}}_{0}\left(t=0\right)=\hat{q},$${\hat{p}}_{0}\left(t=0\right)=\hat{p}.$ From these relations, it follows that there are two other integrals of motion for free particle

(29) $$\hat{a}\left(t\right)=\frac{1}{\sqrt{2}}\left(\hat{q}-\hat{p}t+i\hat{p}\right),\;{\hat{a}}^{†}\left(t\right)=\frac{1}{\sqrt{2}}\left(\hat{q}-\hat{p}t-i\hat{p}\right)$$

with bosonic commutation relations

(30) $$\left[\hat{a}\left(t\right),{\hat{a}}^{†}\left(t\right)\right]=\hat{1}.$$

For t=0, the integrals of motion (29) coincide with the standard creation ${\hat{a}}^{†}$ and annihilation $\hat{a}$ operators of the harmonic oscillator

(31) $$\hat{a}=\frac{1}{\sqrt{2}}\left(\hat{q}+i\hat{p}\right),\;{\hat{a}}^{†}=\frac{1}{\sqrt{2}}\left(\hat{q}-i\hat{p}\right).$$

In view of these properties, one can construct the Fock states |n,t〉 and coherent states of free particle. Using the formalism of coherent and Fock states of harmonic oscillator [8,9], we construct coherent states of free particle. First, we obtain the state |0,t〉 such that it satisfies the condition $\hat{a}\left(t\right)|0,t\rangle =0$ and the Schrödinger equation $\hat{H}|0,t\rangle =i\frac{\partial |0,t\rangle }{\partial t}$ with $\hat{H}={\hat{p}}^{2}/2$. The wave function of the state in the position representation reads

(32) $$\psi_{0}\left(x,t\right)=\frac{1}{\pi^{1/4}}\frac{1}{\sqrt{1+it}}\exp\left(-\frac{x^{2}}{2(1+it)}\right).$$

In the momentum representation, the wave function of the state |0,t〉 reads

(33) $$\psi_{0}\left(p,t\right)=\frac{1}{\pi^{1/4}}\exp\left(-\frac{p^{2}\left(1+it\right)}{2}\right).$$

The coherent state |α,t〉 such that $\hat{a}\left(t\right)|\alpha ,t\rangle =\alpha |\alpha ,t\rangle$ is given by the formula [8,9]

(34) $$|\alpha ,t\rangle =e^{-{\left|\alpha \right|}^{2}/2}e^{\alpha {\hat{a}}^{†}\left(t\right)}|0,t\rangle .$$

The normalized Fock state |n,t〉 is given by the formula

(35) $$|n,t\rangle =\frac{{\left({\hat{a}}^{†}\left(t\right)\right)}^{n}}{\sqrt{n!}}|0,t\rangle .$$

In momentum representation, the Fock state wave function reads

(36) $$\psi_{n}\left(p,t\right)=\psi_{0}\left(p,t\right)\frac{1}{\sqrt{2^{n}n!}}H_{n}\left(p\right),$$

where $\psi_{0}\left(p,t\right)$ is given by Equation (33), and $H_{n}\left(p\right)$ is Hermite polynomial. The coherent state wave function in momentum representation $\psi_{\alpha }\left(p,t\right)$ reads

(37) $$\psi_{\alpha }\left(p,t\right)=\psi_{0}\left(p,t\right)e^{-{\left|\alpha \right|}^{2}/2}\exp\left(-i\alpha \sqrt{2}p+\frac{\alpha^{2}}{2}\right),$$

where $\psi_{0}\left(p,t\right)$ is given by Equation (33).

## 6. Evolution of the Symplectic Tomogram

For the unitary evolution of density operator $\hat{\rho }\left(t\right)$ of the system with Hamiltonian $\hat{H}$, which is given by the evolution operator $\hat{u}\left(t\right)=\exp\left(-it\hat{H}\right)$, we have

(38) $$\hat{\rho }\left(t\right)=\hat{u}\left(t\right)\hat{\rho }\left(0\right){\hat{u}}^{†}\left(t\right).$$

The tomogram w(X|μ,ν,t) which corresponds to the density operator reads

(39) $$w\left(X|\mu ,\nu ,t\right)=\mathrm{Tr}\left(\hat{\rho }\left(t\right)\delta \left(X\hat{1}-\mu \hat{q}-\nu \hat{p}\right)\right).$$

Using the properties of trace of product of operators, we have

(40) $$w\left(X|\mu ,\nu ,t\right)=\mathrm{Tr}\left(\hat{\rho }\left(0\right)\delta \left(X\hat{1}-\mu {\hat{q}}_{H}\left(t\right)-\nu {\hat{p}}_{H}\left(t\right)\right)\right).$$

Here, ${\hat{q}}_{H}\left(t\right)$, ${\hat{p}}_{H}\left(t\right)$ are position and momentum operators in the Heisenberg representation

(41) $${\hat{q}}_{H}\left(t\right)={\hat{u}}^{†}\left(t\right)\hat{q}\hat{u}\left(t\right),\;{\hat{p}}_{H}\left(t\right)={\hat{u}}^{†}\hat{p}\hat{u}\left(t\right).$$

We will consider, following this general approach, the example of free particle motion. In the case of the free particle motion, the Heisenberg operators of position and momentum (m=ℏ=1) are

(42) $${\hat{q}}_{H}\left(t\right)=\hat{q}+\hat{p}t,\;{\hat{p}}_{H}\left(t\right)=p.$$

We obtain the result that the expression for initial tomogram $w_{0}\left(X|\mu ,\nu \right)$ of free particle gives the evolving tomogram expression for time *t* by the change of the variables in the initial tomogram, i.e.,

(43) $$w\left(X|\mu ,\nu ,t\right)=w_{0}\left(X|\mu ,\nu +\mu t\right).$$

If the free motion of the particle is considered as evolution of the oscillator state with the frequency of oscillator becoming equal to zero (the spring of the oscillator instantly disappeared), the initial tomogram of the ground state

(44) $$w_{0}\left(X|\mu ,\nu \right)=\frac{1}{\sqrt{\pi (\mu^{2}+\nu^{2})}}\exp\left(-\frac{X^{2}}{\mu^{2}+\nu^{2}}\right)$$

provides the solution of the kinetic equation for the tomogram in the form of the Gaussian distribution

(45) $$w_{0}\left(X|\mu ,\nu ,t\right)=\frac{1}{\sqrt{\pi (\mu^{2}+{\left(\nu +\mu t\right)}^{2})}}\exp\left(-\frac{X^{2}}{\mu^{2}+{\left(\nu +\mu t\right)}^{2}}\right).$$

Analogously, the tomogram of the coherent state |α,t〉 of the free particle with initial value

(46) $$w_{\alpha }\left(X|\mu ,\nu \right)=\frac{1}{\sqrt{\pi (\mu^{2}+\nu^{2})}}\exp\left(-\frac{{\left(X-{\bar{X}}_{\alpha }\right)}^{2}}{\mu^{2}+\nu^{2}}\right),$$

where X¯α=2(μReα+νImα), is given

(47) $$w_{\alpha }\left(X|\mu ,\nu ,t\right)=\frac{1}{\sqrt{\pi (\mu^{2}+{\left(\nu +\mu t\right)}^{2})}}\exp\left(-\frac{{\left(X-{\bar{X}}_{\alpha }\left(t\right)\right)}^{2}}{\mu^{2}+{\left(\nu +\mu t\right)}^{2}}\right).$$

Here, the mean value ${\bar{X}}_{\alpha }\left(t\right)$ reads

(48) X¯α(t)=2(μReα+(ν+μt)Imα).

For initial Fock state tomogram,

(49) $$w_{0}^{n}\left(X|\mu ,\nu \right)=\frac{1}{\sqrt{\pi (\mu^{2}+\nu^{2})}}\frac{1}{2^{n}n!}\exp\left(-\frac{X^{2}}{\mu^{2}+\nu^{2}}\right){\left(H_{n}\left(\frac{X}{\sqrt{\mu^{2}+\nu^{2}}}\right)\right)}^{2},$$

we have free motion evolution of the tomogram of the Fock state

(50) $$\begin{array}{ccc} & & w_{n}\left(X|\mu ,\nu ,t\right)=\frac{1}{\sqrt{\pi (\mu^{2}+{\left(\nu +\mu t\right)}^{2})}}\frac{1}{2^{n}n!}\exp\left(-\frac{X^{2}}{\mu^{2}+{\left(\nu +\mu t\right)}^{2}}\right)\\ & & \times {\left(H_{n}\left(\frac{X}{\sqrt{\mu^{2}+{\left(\nu +\mu t\right)}^{2}}}\right)\right)}^{2}. \end{array}$$

The optical tomogram $w_{opt}\left(X|\theta ,t\right)$ of discussed states of the free particle motion is given by above expressions (44)–(50), where μ=cosθ, ν=sinθ. For example, the state |0,t〉 has the optical tomogram

(51) $$w_{opt}^{0}\left(X|\theta ,t\right)=\frac{1}{\sqrt{\pi (1+t^{2}\cos^{2}\theta +t\sin2\theta )}}\exp\left(-\frac{X^{2}}{1+t^{2}\cos^{2}\theta +t\sin2\theta }\right).$$

The optical tomogram of the Fock state reads

(52) $$w_{opt}^{n}\left(X|\theta ,t\right)=w_{opt}^{0}\left(X|\theta ,t\right)\frac{1}{2^{n}n!}{\left(H_{n}\left(\frac{X}{\sqrt{1+t^{2}\cos^{2}\theta +t\sin2\theta }}\right)\right)}^{2}.$$

## 7. Qubit States in Probability Representation of Quantum Mechanics

In previous sections, we considered the states of quantum oscillator and free particle motion in probability representation associated with probability distributions of continuous random variable, namely of the position. In this section, we give the construction of simplest quantum system with discrete variables, namely of the spin-1/2 or two–level atom. For this, we recall that there are three pure spin-1/2 states with the state vectors

$${|\psi \rangle }_{3}=\left(\begin{array}{c} 1\\ 0 \end{array}\right){,\;|\psi \rangle }_{1}=\frac{1}{\sqrt{2}}\left(\begin{array}{c} 1\\ 1 \end{array}\right),\;\mathrm{and}\;{|\psi \rangle }_{2}=\frac{1}{\sqrt{2}}\left(\begin{array}{c} 1\\ i \end{array}\right).$$

These states are eigenstates of the spin–projection operators with spin–projection m=+1/2 onto three axis *z*, *x* and *y*. So, three density matrices of these states which are eigenstates of Pauli matrices read

(53) $$\rho_{3}=\left(\begin{array}{cc} 1 & 0\\ 0 & 0 \end{array}\right),\;\rho_{1}=\left(\begin{array}{cc} 1/2 & 1/2\\ 1/2 & 1/2 \end{array}\right),\;\rho_{2}=\left(\begin{array}{cc} 1/2 & -i/2\\ i/2 & 1/2 \end{array}\right).$$

According to Born’s rule [22,23], the arbitrary spin-1/2 state with the density matrix ρ the numbers $p_{n}=\mathrm{Tr}\left(\rho \rho_{n}\right),\;n=1,2,3$ are probabilities to have in the spin-1/2 states spin–projections m=+1/2. It means that the matrix elements $\rho_{jk},\;j,k=1,2$ of the state density matrix ρ are expressed in terms of the probabilities $p_{n}$ as follows [15]:

(54) $$\rho_{11}=p_{3},\;\rho_{12}=\rho_{21}^{*}=\left(p_{1}-1/2\right)-i\left(p_{2}-1/2\right).$$

Thus, three dichotomic probability distributions $\left(p_{1},1-p_{1}\right)$, $\left(p_{2},1-p_{2}\right)$, $\left(p_{3},1-p_{3}\right)$ determine the density matrix of spin-1/2 state. Since the density matrix has only nonnegative eigenvalues the probabilities must satisfy the inequality (following from the Silvester criterion)

(55) $${\left(p_{1}-\frac{1}{2}\right)}^{2}+{\left(p_{2}-\frac{1}{2}\right)}^{2}+{\left(p_{3}-\frac{1}{2}\right)}^{2}\leq \frac{1}{4}.$$

For pure spin state with state vector

$$|\psi \rangle =\left(\begin{array}{c} \psi_{1}\\ \psi_{2} \end{array}\right),\;|\psi_{1}{|}^{2}+{\left|\psi_{2}\right|}^{2}=1,$$

the inequality (55) becomes the equality. This means that the pure states and the mixed states are expressed in terms of three probability distributions of the dichotomic random variables. For a given matrix of Hamiltonian

$$H=\left(\begin{array}{cc} H_{11} & H_{12}\\ H_{21} & H_{22} \end{array}\right),$$

the energy levels of the spin-1/2 states are solutions of the eigenvalue equation H|ψ〉=E|ψ〉 or H|ψ〉〈ψ|=E|ψ〉〈ψ|. Since the density matrix of pure state is expressed in terms of probabilities, the Schrödinger equation has the form of eigenvalue equation for 4–vector with probability components

(56) $$\left(\begin{array}{cccc} H_{11}-E & 0 & H_{12} & 0\\ 0 & H_{11}-E & 0 & H_{12}\\ H_{21} & 0 & H_{22}-E & 0\\ 0 & H_{21} & 0 & H_{22}-E \end{array}\right)\left(\begin{array}{c} p_{3}\\ p_{1}-\frac{1}{2}-i\left(p_{2}-\frac{1}{2}\right)\\ p_{1}-\frac{1}{2}+i\left(p_{2}-\frac{1}{2}\right)\\ 1-p_{3} \end{array}\right)=0.$$

This is probability representation of the Shcrödinger equation for spin-1/2 system, where state is identified with three probability distributions of dichotomic random variables.

The Schrödinger equation for the state evolution has the probability representation which follows from von Neumann equation for density matrix $\rho =\sum_{k}p_{k}|\psi_{k}\rangle \langle \psi_{k}|$, i.e., $\dot{\rho }+i\left[H,\rho \right]=0$ written for probabilities as kinetic equation

(57) $$\left(\begin{array}{c} {\dot{p}}_{3}\\ {\dot{p}}_{1}-i{\dot{p}}_{2}\\ {\dot{p}}_{1}+i{\dot{p}}_{2}\\ -{\dot{p}}_{3} \end{array}\right)=-i\left(\begin{array}{cccc} H_{11} & H_{12} & H_{13} & H_{14}\\ H_{21} & H_{22} & H_{23} & H_{24}\\ H_{31} & H_{32} & H_{33} & H_{34}\\ H_{41} & H_{42} & H_{43} & H_{44} \end{array}\right)\left(\begin{array}{c} p_{3}\\ p_{1}-\frac{1}{2}-i\left(p_{2}-\frac{1}{2}\right)\\ p_{1}-\frac{1}{2}+i\left(p_{2}-\frac{1}{2}\right)\\ 1-p_{3} \end{array}\right),$$

where the matrix elements $H_{jk}$, j,k=1,2,3,4 are expressed in terms of matrix elements of the Hamiltonian matrix $H_{mn}$ (m,n=1,2) as follows:

(58) $$H=\left(\begin{array}{cccc} 0 & -H_{21} & H_{12} & 0\\ -H_{12} & H_{11}-H_{22} & 0 & H_{12}\\ H_{21} & 0 & H_{22}-H_{11} & -H_{21}\\ 0 & H_{21} & -H_{12} & 0 \end{array}\right).$$

So, one has

(59) $$\begin{array}{ccc} & & \left(\begin{array}{c} {\dot{p}}_{3}\\ {\dot{p}}_{1}-i{\dot{p}}_{2}\\ {\dot{p}}_{1}+i{\dot{p}}_{2}\\ -{\dot{p}}_{3} \end{array}\right)=-i\left(\begin{array}{cccc} 0 & -H_{21} & H_{12} & 0\\ -H_{12} & H_{11}-H_{22} & 0 & H_{12}\\ H_{21} & 0 & H_{22}-H_{11} & -H_{21}\\ 0 & H_{21} & -H_{12} & 0 \end{array}\right)\\ & & \times \left(\begin{array}{c} p_{3}\\ p_{1}-\frac{1}{2}-i\left(p_{2}-\frac{1}{2}\right)\\ p_{1}-\frac{1}{2}+i\left(p_{2}-\frac{1}{2}\right)\\ 1-p_{3} \end{array}\right). \end{array}$$

Thus, we obtain the energy levels and evolution equation for qubit states in the probability representation of quantum mechanics where the evolution equation is presented as the linear kinetic equation for the set of three dichotomic probability distributions. The obtained results can be extended to arbitrary qudit states (spin–*j* states, *N*–level atom states). For this, one needs to get expression of the qudit density matrix in terms of probabilities of random variables. It can be done [27] using the following dequantizer—N×N—matrices which have the properties of density matrices presented in the following section.

Let us consider the arbitrary mixed qubit states using the formalism of quantizer–dequantizer operators applying the scheme introduced in Reference [27]. We can check that the four dequantizers which are 2×2 matrices $U^{\left(ji\right)}$, j,i=1,2 have the form

(60) $$\begin{array}{ccc} & & U^{\left(11\right)}=\frac{1}{2}\left(\begin{array}{cc} 1 & 0\\ 0 & 1 \end{array}\right),\;U^{\left(22\right)}=\left(\begin{array}{cc} 1 & 0\\ 0 & 0 \end{array}\right),\\ & & U^{\left(12\right)}=\frac{1}{2}\left(\begin{array}{cc} 1 & 1\\ 1 & 1 \end{array}\right),\;U_{c}^{\left(12\right)}=\frac{1}{2}\left(\begin{array}{cc} 1 & -i\\ i & 1 \end{array}\right). \end{array}$$

The traces of operators $U^{\left(ji\right)}$, $U_{c}^{\left(12\right)}$ are equal to unity, their eigenvalues are nonnegative. The four quantizers are of the form

(61) $$\begin{array}{ccc} & & D^{\left(11\right)}=\left(\begin{array}{cc} 0 & -1+i\\ -1-i & 2 \end{array}\right),\;D^{\left(22\right)}=\left(\begin{array}{cc} 1 & 0\\ 0 & -1 \end{array}\right),\\ & & D^{\left(12\right)}=\left(\begin{array}{cc} 0 & 1\\ 1 & 0 \end{array}\right),\;D_{c}^{\left(12\right)}=\left(\begin{array}{cc} 0 & -i\\ i & 0 \end{array}\right). \end{array}$$

The quantizers and dequantizers satisfy the relations

$$\mathrm{Tr}\left(U^{\left(ii\right)}D^{\left(jj\right)}\right)=\delta_{ij},\;\mathrm{Tr}\left(U_{c}^{\left(12\right)}D_{c}^{\left(12\right)}\right)=1,\;\mathrm{Tr}\left(U_{c}^{\left(12\right)}D^{\left(ij\right)}\right)=0,\;\mathrm{Tr}\left(U^{\left(ij\right)}D_{c}^{\left(12\right)}\right)=0.$$

The density matrix can be presented in the form

$$\rho =D^{\left(11\right)}p_{3}^{\left(11\right)}+D^{\left(22\right)}p_{3}^{\left(22\right)}+D^{\left(12\right)}p_{1}^{\left(12\right)}+D_{c}^{\left(12\right)}p_{2}^{\left(12\right)},$$

where the probabilities $p_{3}^{\left(11\right)}$, $p_{3}^{\left(22\right)},$$p_{1}^{\left(12\right)},$$p_{2}^{\left(12\right)}$ are determined by the dequantizers and density matrix ρ as follows:

$$p_{3}^{\left(11\right)}=\mathrm{Tr}\left(U^{\left(11\right)}\rho \right)=\frac{1}{2},\;p_{3}^{\left(22\right)}=\mathrm{Tr}\left(U^{\left(22\right)}\rho \right),$$

$$p_{1}^{\left(12\right)}=\mathrm{Tr}\left(U^{\left(12\right)}\rho \right),\;p_{2}^{\left(12\right)}=\mathrm{Tr}\left(U_{c}^{\left(12\right)}\rho \right).$$

The density matrix is linear combination of quantizers. The standard parameterization of qudit density matrices by Bloch sphere parameters [65] and relation of it with discussed probability representation of qubit states was studied in Reference [28].

## 8. Qudit State Probability Representation

The formalism of quantizer–dequantizer operators provides the possibility to construct probability representation for arbitrary qudit states. We illustrate this construction on example of qutrit (spin–1 state), three level atom state. We consider the example based on results of qutrit consideration [73]. It is easy to check that nine dequantizers 3×3–matrices $U^{\left(j\right)}$, j=1,2,…,9 can be chosen as follows:

(62) $$U^{\left(1\right)}=\frac{1}{3}\left(\begin{array}{ccc} 1 & 0 & 1\\ 0 & 1 & 0\\ 1 & 0 & 1 \end{array}\right),\;U^{\left(2\right)}=\frac{1}{3}\left(\begin{array}{ccc} 1 & 0 & -i\\ 0 & 1 & 0\\ i & 0 & 1 \end{array}\right),\;U^{\left(3\right)}=\frac{1}{2}\left(\begin{array}{ccc} 1 & 0 & 0\\ 0 & 1 & 0\\ 0 & 0 & 0 \end{array}\right),\;U^{\left(4\right)}=\frac{1}{3}\left(\begin{array}{ccc} 1 & 1 & 0\\ 1 & 1 & 0\\ 0 & 0 & 1 \end{array}\right),\;U^{\left(5\right)}=\frac{1}{3}\left(\begin{array}{ccc} 1 & -i & 0\\ i & 1 & 0\\ 0 & 0 & 1 \end{array}\right),\;U^{\left(6\right)}=\frac{1}{2}\left(\begin{array}{ccc} 1 & 0 & 0\\ 0 & 0 & 0\\ 0 & 0 & 1 \end{array}\right),\;U^{\left(7\right)}=\frac{1}{3}\left(\begin{array}{ccc} 1 & 0 & 0\\ 0 & 1 & 1\\ 0 & 1 & 1 \end{array}\right),\;U^{\left(8\right)}=\frac{1}{3}\left(\begin{array}{ccc} 1 & 0 & 0\\ 0 & 1 & -i\\ 0 & i & 1 \end{array}\right),\;U^{\left(9\right)}=\frac{1}{3}\left(\begin{array}{ccc} 1 & 0 & 0\\ 0 & 1 & 0\\ 0 & 0 & 1 \end{array}\right).$$

All these matrices have unit trace; they satisfy the condition of the density matrices, i.e., ${\left(U^{\left(k\right)}\right)}^{†}=\left(U^{\left(k\right)}\right)$, and eigenvalues of these matrices are nonnegative. The spin–1 states for which these matrices $U^{\left(k\right)}$ are density matrices are not pure states, they are mixed states.

The quantizers $D^{\left(j\right)}$, j=1,2…,9 have the form of the following matrices

(63) $$\begin{array}{ccc} & & D^{\left(1\right)}=\frac{3}{2}\left(\begin{array}{ccc} 0 & 0 & 1\\ 0 & 0 & 0\\ 1 & 0 & 0 \end{array}\right),\;D^{\left(2\right)}=\frac{3}{2}\left(\begin{array}{ccc} 0 & 0 & -i\\ 0 & 0 & 0\\ i & 0 & 0 \end{array}\right),\;D^{\left(3\right)}=2\left(\begin{array}{ccc} 1 & 0 & 0\\ 0 & 0 & 0\\ 0 & 0 & -1 \end{array}\right),\\ & & D^{\left(4\right)}=\frac{3}{2}\left(\begin{array}{ccc} 0 & 1 & 0\\ 1 & 0 & 0\\ 0 & 0 & 0 \end{array}\right),\;D^{\left(5\right)}=\frac{3}{2}\left(\begin{array}{ccc} 0 & -i & 0\\ i & 0 & 0\\ 0 & 0 & 0 \end{array}\right),\;D^{\left(6\right)}=2\left(\begin{array}{ccc} 1 & 0 & 0\\ 0 & -1 & 0\\ 0 & 0 & 0 \end{array}\right),\\ & & D^{\left(7\right)}=\frac{3}{2}\left(\begin{array}{ccc} 0 & 0 & 0\\ 0 & 0 & 1\\ 0 & 1 & 0 \end{array}\right),\;D^{\left(8\right)}=\frac{3}{2}\left(\begin{array}{ccc} 0 & 0 & 0\\ 0 & 0 & -i\\ 0 & i & 0 \end{array}\right),\\ & & D^{\left(9\right)}=\frac{3}{2}\left(\begin{array}{ccc} -2 & -1+i & -1+i\\ -1-i & 2 & -1+i\\ -1-i & -1-i & 2 \end{array}\right). \end{array}$$

One can check that, for the Hermitian matrices $U^{\left(j\right)}$, $D^{\left(j^{\prime }\right)}$, we have the relations

(64) $$\mathrm{Tr}\left(U^{\left(j\right)}D^{\left(j^{\prime }\right)}\right)=\delta_{jj^{\prime }},\;\mathrm{Tr}\left(U^{\left(j\right)}\right)=1.$$

In view of Born’s rule, the numbers $p_{j}=\mathrm{Tr}\left(\rho U^{\left(j\right)}\right)$ are nonnegative dichotomic probabilities, $o\leq p_{j}\leq 1$, and the density matrix ρ of any qutrit state can be presented in the form of linear combination of the quantizers

(65) $$\rho =\sum_{j=1}^{9}p_{j}D^{\left(j\right)},$$

which is parameterized by the dichotomic probabilities $p_{j}$. There exist other dichotomic probability representations of the qudit states, e.g., the qutrit states, where the quantizers are matrices of projectors [74] which can be expressed as linear combinations of the matrices (62). To get the results for arbitrary qudit states with density N×N–matrix $\rho_{jk}$, we introduce other notation for dequantizers and quantizers using the map of the integer numbers $1,2,\ldots ,N^{2}$ onto pairs of integer numbers (jk), where j,k=1,2,…N. For this, we consider the matrices $E_{jk}$, where all the matrix elements are equal to zero, and only one element in *j*-th row and *k*-th column equals to one. These matrices, together with transposed matrices, form the orthogonal basis in the linear space of N×N–matrices. Let us consider the dequantizers which are N×N–matrices:

(66) $$\begin{array}{ccc} & & U^{\left(11\right)}=\frac{1}{N}1,\;U^{\left(jj\right)}=\frac{1}{N-1}\left(1-E_{jj}\right),\;j=2,3,\ldots ,N,\\ & & U^{\left(jk\right)}=\frac{1}{N}\left(E_{jk}+E_{kj}+1\right),\;\\ & & U_{c}^{\left(jk\right)}=\frac{1}{N}\left(1+i\left(-E_{jk}+E_{kj}\right)\right),\;j<k,\;j,k=1,2,\ldots ,N. \end{array}$$

These matrices are hermitian ones and traces of these matrices are equal to unity. They have only nonnegative eigenvalues. These matrices correspond to some density matrices of mixed states of qudits. The quantizer matrices we used are [27]

(67) $$\begin{array}{ccc} & & D^{\left(11\right)}=N\left(1-\left(1-N\right)E_{11}+\frac{1}{2}\left(\left(-1+i\right)\sum_{m<n}^{N}E_{mn}+\left(-i-1\right)\sum_{m<n}^{N}E_{nm}\right)\right),\\ & & D^{\left(jj\right)}=\left(N-1\right)\left(E_{11}-E_{jj}\right),\;j=2,\ldots ,N,\\ & & D^{\left(jk\right)}=\frac{1}{2}N\left(E_{jk}+E_{kj}\right),\;j<k,\;j,k=1,2,N,\\ & & D_{c}^{\left(jk\right)}=\frac{iN}{2}\left(-E_{jk}+E_{kj}\right),\;j<k,\;j,k=1,2,\ldots ,N. \end{array}$$

For qutrit states, these expressions provide the quantizers (63). One has the orthogonality relations

(68) $$\begin{array}{ccc} & & \mathrm{Tr}\left(U^{\left(jk\right)}D^{\left(j^{\prime }k^{\prime }\right)}\right)=\delta_{jj^{\prime }}\delta_{kk^{\prime }},\;\mathrm{Tr}\left(U_{c}^{\left(jk\right)}D_{c}^{\left(j^{\prime }k^{\prime }\right)}\right)=\delta_{jj^{\prime }}\delta_{kk^{\prime }},\\ & & \mathrm{Tr}\left(U_{c}^{\left(jk\right)}D^{\left(j^{\prime }k^{\prime }\right)}\right)=0,\;\mathrm{Tr}\left(U^{\left(jk\right)}D_{c}^{\left(j^{\prime }k^{\prime }\right)}\right)=0,\;j,j^{\prime },k,k^{\prime }=1,2\ldots ,N. \end{array}$$

In view of the Born’s rule, the numbers $0\leq \mathrm{Tr}\left(\rho U^{\left(jk\right)}\right),\mathrm{Tr}\left(\rho U_{c}^{\left(jk\right)}\right)\leq 1$ are the probabilities which satisfy the Silvester criterion for the density matrix ρ. The density matrix ρ can be expressed as the linear combination of the quantizers (67) with the coefficients determined by the probabilities

(69) $$\rho =\sum_{j=1}^{N}D^{\left(jj\right)}p_{3}^{\left(jj\right)}+\sum_{j<k}^{N}D^{\left(jk\right)}p_{1}^{\left(jk\right)}+\sum_{j<k}^{N}D_{c}^{\left(jk\right)}p_{2}^{\left(jk\right)}.$$

Here, the probabilities $p_{3}^{\left(jj\right)},$$p_{1}^{\left(jk\right)}$, $p_{2}^{\left(jk\right)}$ are determined by the dequantizers (66) and the density matrix ρ as follows:

(70) $$\begin{array}{ccc} & & p_{3}^{\left(11\right)}=\frac{1}{N}=\mathrm{Tr}\left(U^{\left(11\right)}\rho \right),\;p_{3}^{\left(jj\right)}=\mathrm{Tr}\left(U^{\left(jj\right)}\rho \right),\;j=2,3,\ldots ,N,\\ & & p_{1}^{\left(jk\right)}=\mathrm{Tr}\left(U^{\left(jk\right)}\rho \right),\;p_{2}^{\left(jk\right)}=\mathrm{Tr}\left(U_{c}^{\left(jk\right)}\rho \right),\;j<k,\;j,k=1,2,\ldots ,N. \end{array}$$

For arbitrary qudit, the construction of density matrix in terms of probabilities of dichotomic random variables can be generalized and given as follows [27]:

(71) $$\rho_{jj}=1-p_{3}^{\left(jj\right)},\;\rho_{jk}=p_{1}^{\left(jk\right)}-\frac{1}{2}+i\left(p_{2}^{\left(jk\right)}-\frac{1}{2}\right),\;j>k.$$

Using other system of the dequantizers, for example, Reference [74], one can obtain different representations of the qudit density matrices in terms of the probabilities. The density *N* × *N*–matrices ρ can be invertibly mapped onto probability $N^{2}$ vector |p〉 with $N^{2}$ components $\rho_{jk}$, where first, second, etc., rows of the density matrix are expressed in terms of the probabilities $p_{1,2}^{\left(jk\right)}$ and $p_{3}^{\left(jj\right)}$. Other aspects of qudit states properties were discussed in References [75,76,77].

The matrix elements $\rho_{11},\;\rho_{12}$ of density matrix of qutrit state according to formulae (71) have explicit form

(72) $$\rho_{11}=1-p_{3}^{\left(11\right)},\;\rho_{12}=\rho_{\left(21\right)}^{*}=p_{1}^{\left(21\right)}-1/2-i\left(p_{2}^{\left(21\right)}-1/2\right).$$

Then, the probabilities $p_{1}^{\left(11\right)},\;p_{3}^{\left(11\right)}$ are expressed through the matrix elements of density matrix and have a view

(73) p3(11)=1−ρ11,p1(21)=Re(ρ12)+1/2.

There is known entropic inequality for probabilities

(74) $$p_{1}^{\left(12\right)}\ln\left(\frac{p_{1}^{\left(12\right)}}{p_{3}^{\left(11\right)}}\right)+\left(1-p_{1}^{\left(12\right)}\right)\ln\left(\frac{1-p_{1}^{\left(12\right)}}{1-p_{3}^{\left(11\right)}}\right)\geq 0.$$

Using the probability representation, we can obtain new entropic inequalities for density matrix elements of qudit state; for example, for qutrit, we have:

(75) Re(ρ12)+1/2lnRe(ρ12)−1/21−ρ11≥Re(ρ12)−1/2ln1/2−Re(ρ12)ρ11.

## 9. Quantum Evolution of Open System in Probability Representation of Density Operator

The evolution of density N×N–matrix for the open system states is given by Gorini–Kossakowski–Sudarshan–Lindblad (GKSL) equation [49,50]

(76) $$\dot{\rho }=-i\left[H,\rho \right]+\frac{1}{2}\sum_{k}\left(\left[V_{k}\rho ,V_{k}^{†}\right]+\left[V_{k},\rho V_{k}^{†}\right]\right).$$

Here, *H* is hermitian Hamiltonian matrix, $V_{k}$ are some matrices. For $V_{k}=0$, the Equation (76) becomes von Neumann equation. Our aim is to rewrite the Equation (76) in the form of linear kinetic equation for the probability vector |p〉. For this, we use example of qubit, and we map matrix ρ onto the vector |ρ〉

(77) $$\begin{array}{ccc} & & |\rho \rangle =\left(\begin{array}{c} \rho_{11}\\ \rho_{12}\\ \rho_{21}\\ \rho_{22} \end{array}\right),\\ & & \rho_{11}=p_{3},\;\rho_{12}=p_{1}-\frac{1}{2}-i\left(p_{2}-\frac{1}{2}\right),\;\rho_{21}=\rho_{12}^{*},\;\rho_{22}=1-p_{3}. \end{array}$$

Here, the parameters $p_{j}$, j=1,2 are dichotomic probabilities. Then, we use the following matrix map which can be checked. Namely, if one has matrix relations for the matrices *A*, ρ, *B* and *C*, ρ, *D*

(78) Aρ=B,ρC=D,

then these relations can be rewritten in the vector form

(79) A|ρ〉=|B〉,C|ρ〉=|D〉.

Here, the matrices A, C read

(80) $$A=A⊗1=\left(\begin{array}{cccc} A_{11} & 0 & A_{12} & 0\\ 0 & A_{11} & 0 & A_{12}\\ A_{21} & 0 & A_{22} & 0\\ 0 & A_{21} & 0 & A_{22} \end{array}\right)$$

and

(81) $$C=1⊗C^{tr}=\left(\begin{array}{cccc} C_{11} & C_{21} & 0 & 0\\ C_{12} & C_{22} & 0 & 0\\ 0 & 0 & C_{11} & C_{21}\\ 0 & 0 & C_{12} & C_{22} \end{array}\right).$$

Using these relations, the GKSL Equation (76) for qubit states can be written in the form of kinetic equation

(82) $$\frac{d}{dt}|p\rangle =M|p\rangle ,$$

where probability vector |p〉, given by (77)

(83) $$|p\rangle =\left(\begin{array}{c} p_{3}\\ p_{1}-\frac{1}{2}-i\left(p_{2}-\frac{1}{2}\right)\\ p_{1}-\frac{1}{2}+i\left(p_{2}-\frac{1}{2}\right)\\ 1-p_{3} \end{array}\right),$$

satisfies this equation, and the matrix M reads

(84) $$M=-i\left(H⊗1-1⊗H^{†}\right)+\sum_{k}\left(V_{k}⊗V_{k}^{*}-\frac{1}{2}V_{k}^{†}V_{k}⊗1-\frac{1}{2}1⊗\left(V_{k}^{\mathrm{Tr}}V_{k}^{*}\right)\right).$$

Equations (82) and (84) are valid for arbitrary qudit. Let us consider as an example the case of a toy-model where the particle with spin-1/2 is interacting only with an environment. The Hamiltonian in this case is chosen to be H=0, the matrices $V_{i},\;i=1,2,\ldots$ are

$$V_{1}=\left(\begin{array}{cc} 1 & 0\\ 0 & 0 \end{array}\right),\;V_{2}=V_{3}=\ldots =0,$$

and the matrix M (84) in explicit form reads

$$M=\left(\begin{array}{cccc} 0 & 0 & 0 & 0\\ 0 & -\frac{1}{2} & 0 & 0\\ 0 & 0 & -\frac{1}{2} & 0\\ 0 & 0 & 0 & 0 \end{array}\right).$$

Solutions of the Equation (84) for probabilities in this case read

$$p_{1}\left(t\right)=\frac{1}{2}+\left(p_{1}\left(0\right)-\frac{1}{2}\right)e^{-t/2},$$

$$p_{2}\left(t\right)=\frac{1}{2}+\left(p_{2}\left(0\right)-\frac{1}{2}\right)e^{-t/2},$$

$$p_{3}\left(t\right)=p_{3}\left(0\right),$$

where $p_{1}\left(0\right)$, $p_{2}\left(0\right)$, $p_{3}\left(0\right)$ are probabilities of spin projection m=+1/2 on axis *x*, *y*, *z* in initial moment of time. In this case, the density matrix of qubit state expressed in terms of probabilities is of the form

(85) $$\begin{array}{ccc} & & \rho (t)=\\ & & \left(\begin{array}{cc} p_{3}\left(0\right) & \left(p_{1}\left(0\right)-\frac{1}{2}\right)e^{-t/2}-i\left(p_{2}\left(0\right)-\frac{1}{2}\right)e^{-t/2}\\ \left(p_{1}\left(0\right)-\frac{1}{2}\right)e^{-t/2}+i\left(p_{2}\left(0\right)-\frac{1}{2}\right)e^{-t/2} & 1-p_{3}\left(0\right) \end{array}\right). \end{array}$$

Let us consider as the next example the case of the second toy-model where qubit is interacting with environment and with a nonzero magnetic field. The magnetic field is directed along *z*–axis. The matrix of Hamiltonian in this case is chosen to be

$$H=\left(\begin{array}{cc} 1 & 0\\ 0 & 0 \end{array}\right),$$

the matrices

$$V_{1}=\left(\begin{array}{cc} 1 & 0\\ 0 & 0 \end{array}\right),\;V_{2}=V_{3}=\ldots =0.$$

The matrix M in this case reads

$$M=\left(\begin{array}{cccc} 0 & 0 & 0 & 0\\ 0 & -i-1/2 & 0 & 0\\ 0 & 0 & i-1/2 & 0\\ 0 & 0 & 0 & 0 \end{array}\right).$$

The solution of the Equation (84) for probabilities in this case are of the form

$$\begin{array}{ccc} & & p_{1}\left(t\right)=\frac{1}{2}+e^{-\frac{t}{2}}\left[\left(p_{1}\left(0\right)-\frac{1}{2}\right)\cos\left(t\right)-\left(p_{2}\left(0\right)-\frac{1}{2}\right)\sin\left(t\right)\right],\\ & & p_{2}\left(t\right)=\frac{1}{2}+e^{-\frac{t}{2}}\left[\left(p_{2}\left(0\right)-\frac{1}{2}\right)\cos\left(t\right)+\left(p_{1}\left(0\right)-\frac{1}{2}\right)\sin\left(t\right)\right],\\ & & p_{3}\left(t\right)=p_{3}\left(0\right). \end{array}$$

## 10. Conclusions

To conclude, we point out the main results of our work. We presented the review of the method of quantizer–dequantizer operators to construct the probability representation of quantum states. It is new formulation of quantum mechanics where the quantum states, both for systems with continuous variables, like harmonic oscillator and free particle, as well as qubit and qudit systems (spin and *N*–level atom) described by discrete variables, are identified with probabilities. The density matrices of the quantum systems are mapped onto the probabilities. An example of the oscillator and a new example of the free particle are explicitly considered. In addition, qubit and qutrit systems are explicitly studied. The GKSL evolution equation for the quantum state density matrices is presented in the form of kinetic equation for probabilities describing the states in quantum mechanics. For continuous variables, the probabilities we use are tomographic probability distributions, which are symplectic or optical tomograms. The problem of quantum free particle motion is considered as example of harmonic oscillator motion in the case of instant change of the oscillator frequency which becomes equal to zero. The tomograms of the coherent states and the Fock states of free particle and their evolution are found explicitly. The free particle coherent state tomograms are found in the form of the Gaussian distributions. The Fock state tomograms of the free particle are obtained and expressed in the form of the probabilities expressed in terms of the Hermite polynomials. These explicit results are new results of the suggested approach. The evolution equation for the probabilities identified with the quantum states of open systems is presented in the form of kinetic equation for qudit states. The examples of two toy-models for qubit open system are explicitly considered. The qubit state example corresponds to the Wootter’s suggestion [5] to construct “probability tables” corresponding to the probability representation of the quantum system. For a hundred years, from the beginning of quantum mechanics, the quantum state notion was determined by wave functions and density matrices. Now, it was shown that there is possibility to determine quantum states and their evolution by probabilities. We point out as the main result of the paper that, in probability representation of quantum mechanics, the states can be identified not only with quasi–probabilities, like the Wigner functions, but also with probability distributions, like the symplectic tomograms found for coherent states of the free particle in the paper.

The new result which is obtained using for qutrit not generalized Bloch sphere parameters of the system state but the probability parameterization is the new entropic inequality for matrix elements of density matrix.

In the work, we presented the review of the quantum mechanics formalism in which the quantum system states are identified with the probability distributions. This formalism is completely equivalent to the usual formalism where the states are identified with the wave functions and the density matrices or with the state vectors and density operators in the Hilbert space. We review the quantum system evolution equation like von Neumann equation written as the evolution equation for the probability distribution identified with system state.

We plan to obtain for qudits other entropic and information equalities and inequalities based on known in probability theory results for the quantum systems using the probability representation of quantum mechanics in future publication.

## Data Availability

Not applicable.
